# Diagnostic Accuracy of EndoFaster® and Narrow-Band Imaging Endoscopy in Patients with Impaired Gastric Acid Secretion: A Real-Time Prospective Study

**DOI:** 10.1155/2021/6616334

**Published:** 2021-03-20

**Authors:** M. Cazzato, G. Esposito, G. Galli, E. Pilozzi, E. Lahner, V. D. Corleto, A. Zullo, E. Di Giulio, B. Annibale

**Affiliations:** ^1^Department of Medical-Surgical Sciences and Translational Medicine, Sant'Andrea Hospital, Sapienza University of Rome, Italy; ^2^Department of Pathology, Sant'Andrea Hospital, Sapienza University of Rome, Italy; ^3^Gastroenterology and Digestive Endoscopy, “Nuovo Regina Margherita” Hospital, Rome, Italy

## Abstract

**Background:**

EndoFaster® analyzes gastric juice in real time during gastroscopy allowing the detection of hypo-achlorhydric conditions, like corpus atrophic gastritis. Narrow-band imaging (NBI) endoscopy allows to accurately detect and perform target biopsies in areas of intestinal metaplasia, a histological change often associated to corpus atrophic gastritis.

**Aims:**

To compare the diagnostic accuracy of EndoFaster® with histological evaluation for corpus atrophic gastritis through high-resolution (HR) NBI targeted biopsies.

**Methods:**

Prospective study on consecutive adult patients undergoing gastroscopy between April and November 2018. Patients in therapy with proton pump inhibitors, previous gastric surgery, and/or known gastric neoplasia were excluded. At the beginning of gastroscopy, gastric juice was aspirated and analyzed by EndoFaster® in 15 seconds. Endoscopists were blinded to the report of EndoFaster®. Evaluation of gastric mucosa in HR-white light was firstly performed, then with HR-NBI allowing to perform targeted biopsies on areas suspected for intestinal metaplasia; otherwise, biopsies were performed according to the updated Sydney System protocol and sent for histopathological evaluation.

**Results:**

Overall, 124 patients were included [64% F; 56 (18-85) years]. Corpus atrophic gastritis was present in 41.9% of patients. EndoFaster® showed an accuracy for corpus atrophic gastritis diagnosis, compared to histopathological evaluation as gold standard, of 87.1% and a sensitivity, specificity, PPV, and NPV of 78.8%, 93.1%, 89.1%, and 85.9%, respectively. pH showed a positive correlation with the severity score of atrophy (*r* = 0.67, 95% CI: 0.73-0.81, and *p* < 0.0001). EndoFaster® allowed to diagnose corpus atrophic gastritis in 3.7% of patients negative to NBI (corpus atrophic gastritis without intestinal metaplasia).

**Conclusion:**

EndoFaster® seems a promising tool to diagnose corpus atrophic gastritis. The evaluation of hypo-achlorhydria during gastroscopy can address bioptic sampling in corpus atrophic gastritis patients without intestinal metaplasia.

## 1. Background

Corpus atrophic gastritis (CAG) and intestinal metaplasia (IM) are precancerous conditions since they represent the basis for the development of dysplasia and adenocarcinoma. Therefore, their timely diagnosis represents the best way to reduce cancer-related mortality [1, 2].

CAG is characterized by the reduction or loss of the original gastric glands, which can be replaced by fibrosis, IM, or pseudo-pyloric metaplasia [3]. These conditions can be consequent to a chronic *Helicobacter pylori* (*H*. *pylori*) infection or they can arise in the context of autoimmune gastritis [4–6]. When these alterations involve the oxynthic mucosa, atrophy induces a lack of gastric acid production resulting in gastric hypochlorhydria/achlorhydria which can lead to iron and/or cobalamin malabsorption and possibly anemia [4, 7, 8]. Thus, the importance of making a correct diagnosis of CAG is crucial and is currently based on gastric biopsies performed during an esophagogastroduodenoscopy (EGD), in accordance with the updated Sydney System [9]. However, the standard EGD, performed in white light endoscopy (WLE), has important diagnostic limitations since gastric mucosal changes are often not easily recognizable morphologically and chromatically.

In recent years, new endoscopic techniques have been developed in order to improve the diagnostic power of the basic endoscopy, in particular, electronic chromoendoscopy. In this setting, several studies have been conducted on high-resolution narrow-band imaging (HR-NBI), a technique of electronic chromoendoscopy, which is able to detect IM during endoscopic examination allowing to perform targeted biopsies in areas suspected for IM often associated with CAG [10–12].

However, EGD with biopsy sampling is not a good screening tool for CAG in areas with low incidence because it is an expensive and demanding method [13]. A quick and economic tool would be needed in order to preselect patients to be screened. In this setting, an important role could be played by the analysis of the gastric juice which represents a sensitive marker of reduced acid secretion. Therefore, during the endoscopic examination, the analysis of gastric juice may indirectly predict the presence/absence of CAG [14–16]. This means that in the suspicion of hypochlorhydria, it would be necessary to perform gastric biopsies in all patients; however, this is often not feasible in clinical practice as it would lead to a strong increase in times and costs.

The problem could be solved by predicting the presence of hypochlorhydria during the endoscopic examination. For this purpose, NISO Biomed EndoFaster® 21-42 (EF) was developed. It represents a medical device able to analyze gastric juice automatically and in real time, through EGD. The operating principle of the device is based on determining pH of the gastric juice, allowing the detection of a hypochlorhydric condition. The device is also able to indirectly assess the possible presence of *H*. *pylori* infection by defining the ammonium concentration in gastric juice.

Thanks to its easy use and low cost; this device could represent an important mass screening method able to select patients who need a more accurate investigation by EGD with biopsies.

Previous studies compared EF to EGD in high-resolution- (HR-) WLE proving its high accuracy for the diagnosis of hypochlorhydria [16–19]. However, no study compared the accuracy of EF associated to the HR-NBI examination with targeted biopsies in a population with CAG or dyspepsia.

For this reason, we focused on patients with a high chance to have hypochlorhydric conditions without taking into consideration the results about *H*. *pylori* infection because it has been already demonstrated.

The study is aimed at determining the accuracy of EF in assessing the presence of normal or hypochlorhydric status during gastroscopy through HR-NBI targeted biopsies, compared to histopathological evaluation as gold standard in patients with suspicion of CAG.

## 2. Methods

### 2.1. Study Population

A prospective study was conducted on 161 consecutive adult patients (63% female; median age 60, range 18-85, years) who underwent EGD for clinical suspicion of CAG, in a single unit of Digestive Endoscopy (University Hospital Sant'Andrea, Rome) in the period between April and November 2018.

**Figure 1:**
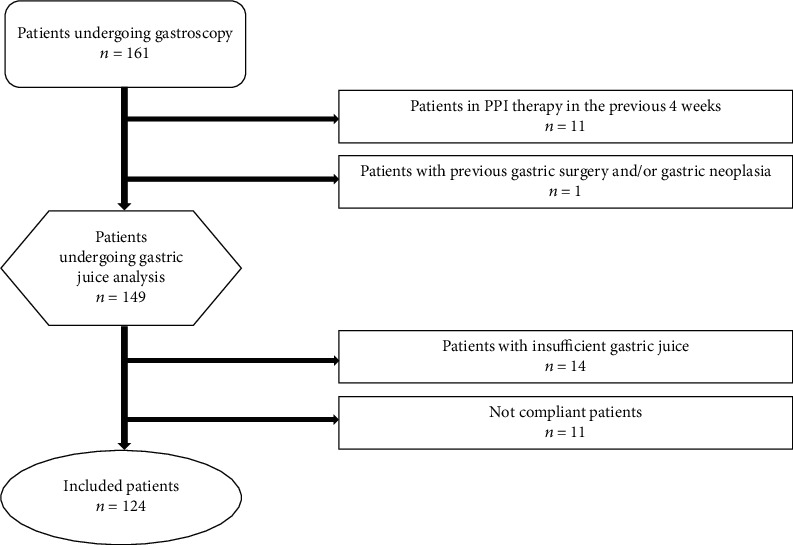
Flow chart of the study population.

Exclusion criteria were proton pump inhibitor therapy in the previous 4 weeks, previous gastric surgery, and/or known gastric neoplasia. Patients were also excluded when they had insufficient gastric juice concentration or when they were not compliant to the endoscopic examination (Figure 1).

The reporting of this study was performed following the Standards for Reporting of Diagnostic Accuracy Studies (STARD) guidelines [20]. All patients signed an informed written consent, and the study was approved by local Ethical Committee.

### 2.2. Endoscopy

All patients underwent a conventional upper endoscopy under sedation with benzodiazepine using Olympus video gastroscope (GIF–H185 or GIF–H160). The EGD was started with the intubation of the esophagus, and as soon as the stomach was reached, a sample of gastric juice (2 ml) was aspirated and analyzed by EF in real time. During the intubation, a special attention was paid to not aspirate until the stomach was reached because of the risk to aspirate other liquids different from gastric juice to avoid biased gastric juice evaluation. After aspiration of gastric juice, the endoscopic procedure continued by an accurate evaluation of gastric mucosa first in HR-WLE and subsequently in HR-NBI. The assessment of the gastric mucosa by electronic chromoendoscopy allowed to perform targeted biopsies where the suspicion of IM was present. In case of normal gastric mucosa at NBI examination, biopsies were performed randomly using the updated Sydney System protocol [9]: 2 biopsies of the antrum (within 3 cm from the pylorus, large and small curve), 1 biopsy of the incisura, in the same vial, and 2 biopsies of the gastric body (small and large curve) in another vial.

### 2.3. Analysis of Gastric Juice

As soon as the stomach was reached, a sample of gastric juice (2 ml) was aspirated and analyzed in real time within 15 seconds allowing to identify pH value. When the minimum quantity of gastric juice was obtained, EF made a sound to let the operator know that the 2 ml level was reached. In case of low quantity of gastric juice, the endoscopist could decide to apply a dilution, pressing a button on the device that provided an additional dilution allowing to perform the analysis anyway.

The operator could have been informed directly about the results by a voice system and by printing a receipt with data about pH. This would allow the endoscopist to obtain important information in real time during the endoscopic procedure. During the procedures for this study, the voice system was disabled, so that the endoscopist was not aware of the result of the device in order to do not affect the inspection of the stomach and the biopsy sampling. The printed receipt was handled by the assisting nurses.

If the concentration of gastric juice was too small (less than 1.5 ml of gastric juice), the device automatically interrupted the procedure and the patient was not included in the study. Depending on the pH value measured, the device is able to distinguish three different frames related to pH: normal gastric acidity (pH < 3), mild hypochlorhydria (3 ≤ pH < 4.5), and severe hypochlorhydria (pH ≥ 4.5).

### 2.4. Histopathology

The biopsy specimens were fixed in formalin and treated routinely. The gastric mucosa sections were stained with hematoxylin-eosin for routine examination. The histopathological evaluation was expressed by an expert pathologist (EP), according to updated Sydney System [9] and used as gold standard for the diagnosis of CAG [21, 22]. According to this classification, the atrophy of the gastric mucosa was defined as a focal or complete disappearance or substitution of the oxyntic glands by pyloric or IM. This variable was classified by a four-degree scale represented by the absence of substitution (score 0), mild substitution (score 1), moderate (score 2), or severe substitution (score 3). The atrophy of the antrum mucosa was defined as a focal or complete disappearance of the antral glands or their replacement with IM.

### 2.5. Serological Assays of Pepsinogen I

Serum pepsinogen I (PG I) is a marker of gastric mucosal atrophy and reflects the functional status of gastric mucosa [23, 24]. This evaluation was performed in a subgroup of 53 patients of whom the serum was available. In these patients, PG I was assessed by ELISA test using a commercial kit (Biohit Oyj, Helsinki, Finland; normal value = 30 − 120 *μ*g/l).

### 2.6. Statistical Analysis

Standard descriptive statistics were expressed as median and range or absolute counts and percentages. Diagnostic accuracy was expressed by using sensitivity, specificity, positive predictive value (PPV), and negative predictive value (NPV). Statistical correlations between pH and grade of atrophy and pH and PG I were made using Spearman rank correlation. Statistical analyses were performed using a dedicated software (MedCalc Software, Mariakerke, Belgium, version 17.4).

## 3. Results

Overall, 124 adult outpatients [64% F; median age 56 (range 18-85) years] were included in the study.

Demographics and clinical characteristics of the included patients are detailed in Table 1.

### 3.1. Histopathology

Histopathological evaluation showed OLGA 0, I, II, III, and IV in 69 (55.6%), 11 (9.0%), 37 (29.8%), 4 (3.2%), and 3 (2.4%) patients, respectively. Eighty-one (65.4%) patients were OLGIM 0, 17 (13.7%) were OLGIM I, 20 (16.1%) were OLGIM II, 4 (3.2%) were OLGIM III, and 2 (1.6%) were OLGIM IV. CAG was detected in 52 (41.9%) patients of whom 12 (23.1%) presented mild atrophy, 4 (7.7%) moderate atrophy, and 36 (69.2%) severe atrophy. *H*. *pylori* infection was present in 16 (12.9%) patients.

### 3.2. EndoFaster®

EF showed hypochlorhydria in 46 patients (37.1%) and normal pH in 78 patients (62.9%).

According to EF results, 62.9%, 1.6%, and 35.5% of patients presented a normal gastric acidity (pH < 3), mild hypochlorhydria (3 ≤ pH < 4.5), and severe hypochlorhydria (pH ≥ 4.5), respectively.

### 3.3. EndoFaster® vs. Histopathology

**Figure 2:**
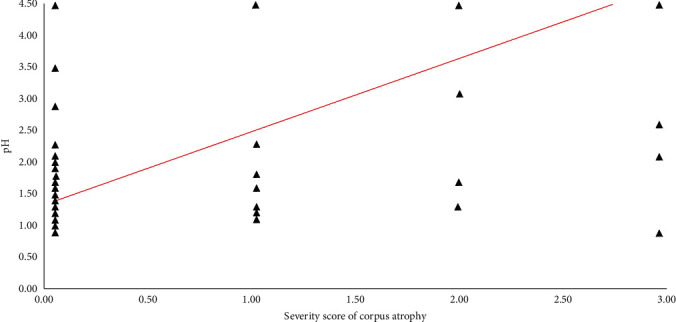
Positive correlation between pH values and histological severity score of atrophy expressed according to updated Sydney System. 0 = absence; 1 = mild; 2 = moderate; 3 = severe (*r* = 0.67; 95% CI: 0.7-0.8; *p* < 0.0001).

Overall, of the 46 patients with hypochlorhydria detected by EF, 41 had a hypochlorhydric condition confirmed by histopathology.  Table 2 shows the comparison between pH measurement and gastric mucosal histology. Considering the 41 (33%) patients with hypochlorhydria confirmed by histology, 7 (17%) of them presented only CAG without IM and the other 34 (83%) patients had CAG with IM. Of the 7 patients without IM, 6 (85.7%) presented severe hypochlorhydria and 1 (14.3%) had mild hypochlorhydria. All of the 34 patients with IM presented severe hypochlorhydria. Compared to histological examination in assessing CAG, EF showed an accuracy, sensitivity, specificity, PPV, and NPV of 87.1%, 78.8%, 93.1%, 89.1%, and 85.9%, respectively. A total of 11 (21.2%) patients showed normal acidity with histological presence of corporal atrophy (pH-false negative), while 5 (6.9%) patients showed hypochlorhydria with histological absence of corporal atrophy (pH-false positive). Features of these patients are described in Table 3. Most pH-false-negative patients (66.7%) had mild atrophic changes, a condition that might not be able to substantially reduce acid secretion. Considering the pH-false-positive patients, 3 of 5 patients had chronic superficial *H*. *pylori*-related corpus-involving gastritis, a condition associated with reduced gastric acid secretion. A positive correlation was found between the pH values and the severity scores of atrophy (*r* = 0.67; 95% CI: 0.7-0.8; *p* < 0.0001Figure 2).

### 3.4. EndoFaster® vs. Pepsinogen I

**Figure 3:**
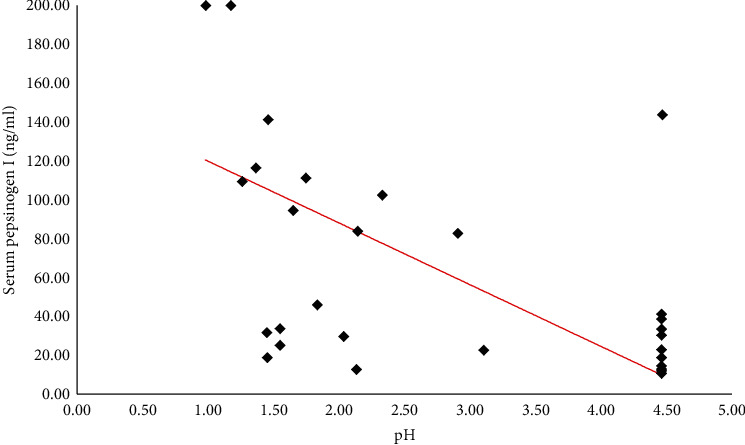
Negative correlation between pH values and pepsinogen I (*r* = −0.67; 95% CI: (-0.8)-(-0.5); *p* < 0.0001). Pepsinogen I values are expressed in microgram per liter. Normal value = 30 − 120 *μ*g/l.

Considering in a subset of 53 patients, the relationship between pH and PG I values, an inverse correlation was found (*r* = −0.67; 95% CI: (-0.8)-(-0.5); *p* < 0.0001Figure 3).

### 3.5. HR-NBI vs. Histopathology

The evaluation of gastric mucosa by HR-NBI was performed in a subgroup of 108 patients (67% F; median age 58 (range 18-85) years). IM of the corpus mucosa was detected in the 39% of them. HR-NBI showed an accuracy of 90% and a sensitivity, specificity, PPV, and NPV of 90%, 89.7%, 83.7%, and 93.8%, respectively, for the diagnosis of corpus IM, compared to histopathology as gold standard.

### 3.6. EndoFaster® vs. HR-NBI

When comparing EF results with those obtained by HR-NBI, a concordance was documented in 93 (86%) patients. In the remaining 15 (14%) patients, HR-NBI showed diagnostic superiority according to the histopathology and observing IM in 11 (74%) patients without evidence of hypochlorhydria at the EF examination. In 3.7% of patients not presenting corpus IM at HR-NBI, EF showed a condition of hypochlorhydria. At histopathology, all these patients presented atrophy of the corpus mucosa without IM.

### 3.7. EndoFaster® plus HR-NBI vs. Histopathology

During upper endoscopy, gastric mucosa was evaluated with the combined use of EF plus HR-NBI in a total of 108 patients (67% F; median age 58 (range 18-85) years). Histological examination revealed the presence of IM and/or CAG in 47% of patients. This finding was confirmed by EF plus HR-NBI evaluation showing that 90% of those patients presented hypochlorhydria and/or endoscopically recognizable metaplasia. Compared to histological examination as gold standard in assessing corpus atrophy and/or IM, the combined use of EF plus HR-NBI showed an accuracy of 90.7% and a sensitivity, specificity, PPV, and NPV of 90.2%, 91.2%, 90.2%, and 91.2%, respectively.

## 4. Discussion

This is the first study which evaluated the role of EF in combination with HR-NBI targeted biopsies, capable of providing an additional support in selecting patients at high suspicion for CAG.

Previous studies already showed a direct correlation between gastric hypochlorhydria and precancerous conditions by comparing the analysis of gastric juice with the histological evaluation conducted on random biopsies. However, so far, no studies considered the additional role of HR-NBI target biopsies in allowing the recognition of patients at risk for CAG [17, 18, 25].

Our work demonstrated a high accuracy of EF in the diagnosis of gastric precancerous conditions with low discrepancy results between HR-NBI and EF (only 14% of cases). In these patients, we found a superiority of HR-NBI in detecting precancerous conditions in 11 patients (74%), documented by histopathological assessment. In 4 patients with normal HR-NBI aspect, EF showed lower grades of hypochlorhydria, histologically confident with mild atrophy. Considering that the early stage of precancerous conditions may not be recognized endoscopically, EF could be a key factor for the identification of atrophy in the absence of IM.

According to the achieved results, EF may be taken into consideration as a support tool to identify patients at risk for CAG requiring an appropriate bioptic mapping of gastric mucosa for definite diagnosis.

Although EGD with biopsies is a highly effective method, it is rarely used as a tool in screening programs as it is an invasive and expensive examination. In this context, an important role could play EF which could provide important information on the gastric function in a few seconds and with inexpensive costs. This could allow to direct the complete endoscopic examination, comprehensive of adequate biopsy sampling, only in patients with impaired gastric acid production.

Our study showed that EF has a considerable high specificity and PPV in detecting gastric hypo/achlorhydric conditions, permitting to select patients at high risk of CAG where biopsies are needed. On the other hand, considering the sensitivity and the NPV, it should be emphasized that 8 of the 11 false-negative patients at the EF test had a mild grade of atrophy which is probably associated to a slight increase of the gastric pH. This condition could be due to the persistence of a sufficient mass of residual parietal cells which are still able to carry out their physiological function; probably in this context, EF is not able to detect the slight change in gastric pH.

Concerning the subgroup of patients investigated by HR-NBI, a high accuracy (90%) in detecting histological IM was shown, in line with other studies in which the increased diagnostic accuracy of HR-NBI in identifying the IM was reported [11, 12].

EF could be useful in identifying other gastric hypochlorhydric conditions that differ from CAG such as proton pump inhibitor (PPI) therapy [26] and chronic *H*. *pylori*-related gastritis involving the corpus mucosa. In fact, in the present study, 60% of the false-positive patients at EF examination showed this histopathological finding. Previous results with EF never focused on the value of hypochlorhydria as a possible clue of *H*. *pylori*-related corpus gastritis, but only on its association with preneoplastic conditions of the stomach. It has been reported that corpus gastritis related to *H*. *pylori* infection can increase the pH of gastric juice due to the functional impairment of the gastric acid secretion by parietal cells [5, 16], and this was here confirmed.

According to the results achieved in our study, EF could improve the diagnostic performance of standard gastroscopy in the detection of precancerous conditions. An important additional contribution could be made by the combined use of EF plus HR-NBI when this resource is available on the equipment. Our work showed that the combined use of the two methods allowed to obtain an accuracy, sensitivity, specificity, PPV, and NPV greater than 90%. The combined use of these devices could provide important information quickly and without additional costs, and it would allow to overcome some limitations related to the single use of each of them.

However, considering the limited diffusion of HR-NBI to relative few specialized endoscopy centres, the methodology here presented may not be widely applicable at this moment. This is the main limitation of this work. However, electronic chromoendoscopy nowadays is spreading in most endoscopic units due to its ability to improve the detection of IM during endoscopic examination; probably, in the next future, many endoscopic units will be equipped with this tool. The overall rate of *H*. *pylori* infection was relatively low (12.9%) in this study because several included patients were previously treated with eradication therapy, so the results on *H*. *pylori* obtained through EF were not taken into consideration. Another limit was that the EF analysis cannot be performed when an insufficient amount of gastric juice is obtained, thus representing a technical limit of the device. In fact, this methodology was not applied to 14 patients diagnosed with CAG, a condition which is able to significantly reduce gastric acid secretion, especially in the advanced stages [13] for severe oxyntic mucosa damage.

## 5. Conclusion

EF seems a promising tool to identify gastric hypo/achlorhydric conditions allowing to recognize, in a few seconds, patients at high suspicion for CAG where a timely diagnosis with adequate bioptic mapping is necessary.

## Figures and Tables

**Table 1:** Baseline characteristics of the 124 included patients.

Baseline characteristics	*n* (%)
Female	80 (64.5)
Median age (range), years	56 (18-85)
Smoking	26 (20.9)
Alcohol (≥1 U/die)	18 (14.5)
Therapy with ASA/NSAID	33 (26.6)
Previous treatment of *H*. *pylori*	44 (35.4)
Cured of infection	19 (15.3)
First-degree relatives with gastric cancer	9 (7.2)
Main indications to gastroscopy	
Clinical suspicion of CAG	58 (46.8)
Not investigated dyspepsia	38 (30.6)
Dyspepsia+gastroesophageal reflux disease	28 (22.6)

ASA: acetylsalicylic acid; NSAID: nonsteroidal anti-inflammatory drugs; CAG: corpus atrophic gastritis.

**Table 2:** pH spot measurements (by EndoFaster®) and gastric mucosal histopathology in the 124 included patients.

	Corpus atrophy^∗^ *n* (%)	No corpus atrophy^∗∗^ *n* (%)	*p*
pH < 3	11 (14.1)	67 (85.9)	<0.0001
≤3 pH < 4.5	1 (50.0)	1 (50.0)	
pH ≥ 4.5	40 (90.9)	4 (9.1)	<0.0001

^∗^Atrophy detected only in the gastric body OR both in the gastric body plus antrum (pan-atrophy). ^∗∗^Atrophy detected only in the gastric antrum OR absence of atrophy.

**Table 3:** Features of pH-false-negative patients (patients with normal acidity and histological presence of corpus atrophy) and pH-false-positive patients (patients with hypochlorhydria and histological absence of corpus atrophy).

	Characteristics	*n* (%)	pH
3a. pH-false-negative patients (*n* = 11)	CAG		
	(i) Mild atrophy	8 (66.7)	1.6, 0.9-2.3°
	(ii) Moderate atrophy^∗^	1 (8.3)	1.7
	(iii) Severe atrophy	2 (16.7)	2.1, 2.6
	^∗^pts with *H*. *pylori* infection	1 (8.3)	
	Previously treated infection of *H*. *pylori*	8 (66.7)	
	Smoking	3 (25.0)	
	Alcohol (≥1 U/die)	5 (41.7)	
	Therapy with ASA/NSAID	3 (25.0)	

3b. pH-false-positive patients (*n* = 5)	Chronic superficial *H*. *pylori-*related corpus gastritis	3 (60.0)	3.5-4.5
	Chronic gastritis (negative HP)	2 (40.0)	≥4.5
	Previously treated *H*. *pylori* infection	1 (20.0)	
	Smoking	0 (0.0)	
	Alcohol (≥1 U/die)	3 (60.0)	
	Therapy with ASA/NSAID	0 (0.0)	

°Median, range. ASA: acetylsalicylic acid; NSAID: nonsteroidal anti-inflammatory drugs; CAG: corpus atrophic gastritis.

## Data Availability

The data used to support the findings of this study are available from the corresponding author upon request.

## Additional Points

*Key Summary*. Current knowledge: (i) corpus atrophic gastritis (CAG) and intestinal metaplasia (IM) represent gastric precancerous conditions requiring timely diagnosis. (ii) High-resolution narrow-band imaging (HR-NBI) has been shown to improve the real-time detection of IM, a histological change often associated to CAG. (iii) CAG strongly correlates with an increase of pH of the gastric juice, and the analysis of gastric juice should indirectly predict the presence/absence of CAG. (iv) NISO Biomed EndoFaster® (EF) is a medical device which automatically analyzes gastric juice in real time during gastroscopy determining the pH of gastric juice and allowing the detection of hypo-achlorhydric conditions. Key findings of this study: (i) EF seems a promising tool to identify CAG allowing to obtain, in few seconds, reliable information about the patients' gastric juice. (ii) This is the first study that combined the use of EF with NBI targeted biopsies showing a high accuracy in the diagnosis of precancerous conditions of the stomach. (iii) EF permitted to diagnose CAG in patients without IM who would not have been diagnosed by NBI, showing the usefulness of this device in endoscopic practice.
